# Thermo-Viscoelastic Response of 3D Braided Composites Based on a Novel FsMsFE Method

**DOI:** 10.3390/ma14020271

**Published:** 2021-01-07

**Authors:** Jun-Jun Zhai, Xiang-Xia Kong, Lu-Chen Wang

**Affiliations:** 1 Department of Aircraft Design, North China Institute of Aerospace Engineering, Langfang 065000, China; wangluchen0721@nciae.edu.cn; 2 Department of Engineering Mechanics, Harbin University of Science and Technology, Harbin 150080, China; 3 Department of Material Engineering, North China Institute of Aerospace Engineering, Langfang 065000, China; kongxx@nciae.edu.cn

**Keywords:** temperature, braided composite, three-scale, thermo-viscoelastic behavior

## Abstract

A homogenization-based five-step multi-scale finite element (FsMsFE) simulation framework is developed to describe the time-temperature-dependent viscoelastic behavior of 3D braided four-directional composites. The current analysis was performed via three-scale finite element models, the fiber/matrix (microscopic) representative unit cell (RUC) model, the yarn/matrix (mesoscopic) representative unit cell model, and the macroscopic solid model with homogeneous property. Coupling the time-temperature equivalence principle, multi-phase finite element approach, Laplace transformation and Prony series fitting technology, the character of the stress relaxation behaviors at three scales subject to variation in temperature is investigated, and the equivalent time-dependent thermal expansion coefficients (TTEC), the equivalent time-dependent thermal relaxation modulus (TTRM) under micro-scale and meso-scale were predicted. Furthermore, the impacts of temperature, structural parameters and relaxation time on the time-dependent thermo-viscoelastic properties of 3D braided four-directional composites were studied.

## 1. Introduction

Three-dimensional (3D) braided composites are increasingly being used in the manufacturing of structural parts due to their incomparable advantages compared with traditional laminated composites in recent years. In order to ensure the safety and reliability in use of the 3D braided composite, the mesoscopic configuration modeling and mechanical performance prediction under multiple working conditions [1,2,3,4,5,6] of 3D braided composites have recently received considerable attention by many scholars. It is well known that the main body of the braided composites is matrix material, and polymer as matrix materials shows obvious viscoelasticity in composites. Hence, the braided composites will also exhibit considerable viscoelastic behavior. With the rapid development of science and technology, various approaches [7,8,9,10,11,12,13,14,15,16,17] have been devised to deal with the viscoelastic problems of 3D braided composites. Liu et al. [7] and Li et al. [8] studied the creep behavior of resin-based 3D braided composites by tensile creep test, and discussed the influence of braided structure and fiber volume fraction on the creep behavior of materials. Priyank et al. [9] set up a three-dimensional micromechanical analytical model based on the classical laminate theory, and studied the equivalent elastic and viscoelastic properties of a 3D braided composites. In addition, the multi-scale method was extensively to predict the viscoelastic behaviors of 3D braided composites. Yuan et al. [10] used the multi-scale method to study the viscoelastic properties of 3D braided composites, and gave the variation law of viscoelastic properties with process parameters. Cai et al. [11] studied the viscoelasticity of 3D braided composites based on the interior cell model and yarn model, and discussed the influence of braiding parameters on the viscoelasticity behavior. Mourid et al. [12] developed an analytical homogenization technique to predict the viscoelastic behavior of 3D braided composites by extending the elastic homogenization frame and coupling the Laplace–Carson transforms. Zhou et al. [13] studied the elastic constants and creep performance by a multiple scale viscoelastic model for the 3D braided composites. Liu et al. [14] studied the viscoelastic behaviors of 3D braided composites based on a yarn solid model and mechanics of structure genome plate model. Jia et al. [15] analyzed the nonlinear viscoelastic responses and damage mechanisms of 3D orthogonal composite under quasi-static tensile loading based on a micro/meso-scale repeated unit cells model. Tate et al. [16] presented the viscoelastic effects on fatigue behavior of 3D carbon/epoxy braided composites based on the experimental methods. Zhai et al. [17] proposed a multi-scale calculation method to study the stress relaxation response of 3D braided composites at three scales, and characterized the viscoelastic properties at different scales.

Thermoviscoelasticity is an important thermo mechanical property for failure and failure of resin matrix composites, which is often used in the long-term and variable temperature environment. Hwang et al. [18] predicted the stress and deformation histories of notchless and notched graphite/epoxy composites under mechanical and thermal loads based on finite-element formula. Muddasani et al. [19] presented some experimental works and finite element analyses of nonlinear thermo-viscoelastic behaviors for multilayered composites. Li et al. [20] established a 3D incremental viscoelastic constitutive model, the residual stresses and the curing deformation of T-shaped composite structures during curing studied. Similarly, it is particularly significant to study the thermo-viscoelastic behaviors before the 3D resin-based braided composite can be confidently used in the primary structures. Seifert et al. [21] studied the time-temperature dependent material properties of 3D braided composite material based on experimental and numerical methods. Hirsekorn et al. [22] obtained the viscoelastic behavior of 3D braided composites from the dependence of component on temperature and degree of cure by a homogenization strategy. Cai et al. [23] reported the thermo-viscoelastic properties of 3D braided composites in different temperature states based on the three-cell model. Sun et al. [24] predicted the dynamic thermo-viscoelasticity response of 3D braided composites to alternating stress load at room temperature, and studied the relationship between the complex compliance and angular frequency based on the mesoscopic representative unit cell (RUC).

These great results are of great significance to explore the thermo-viscoelastic performance of 3D braided composites, but the research on the thermo-viscoelasticity behavior of 3D braided composites are mostly based on the meso-scale RUC and the time-temperature-dependent stress distributions have not received special attention. The time-temperature-dependent mechanical behavior correlation at different scales is not clear, some alternative numerical methods are necessary. To address this drawback, this paper presented a homogenization-based five-step multi-scale finite element (FsMsFE) framework for handling the thermo-viscoelasticity behaviors of 3D braided four-directional composites based on the correspondence principle and time-temperature equivalence principle. In the micro-scale, each kind of fiber and matrix is modeled separately. In the meso-scale, the reinforcement structure is modeled and yarns composed of several thousand of fibers are regarded as homogeneous material. In the macro-scale, the structure is modeled as homogeneous material. Based upon the Prony series fitting, the characters of the thermal stress relaxation at three scales subject to variation in temperature are investigated, and the equivalent time-dependent thermal expansion coefficients (TTEC), the equivalent time-dependent thermal relaxation modulus (TTRM) in micro/meso-scale were predicted. Furthermore, the effects of braiding parameters, relaxation time and temperature on the time-dependent thermo-viscoelastic properties of 3D braided four-directional composites were analyzed. This research could also be extended to the study of thermo-viscoelasticity behaviors of 3D multidirectional polymer-based braided composites or the inhomogeneous materials with periodic structures.

## 2. Multi-Scale Coupling for 3D Braided Composites

**Figure 1:**
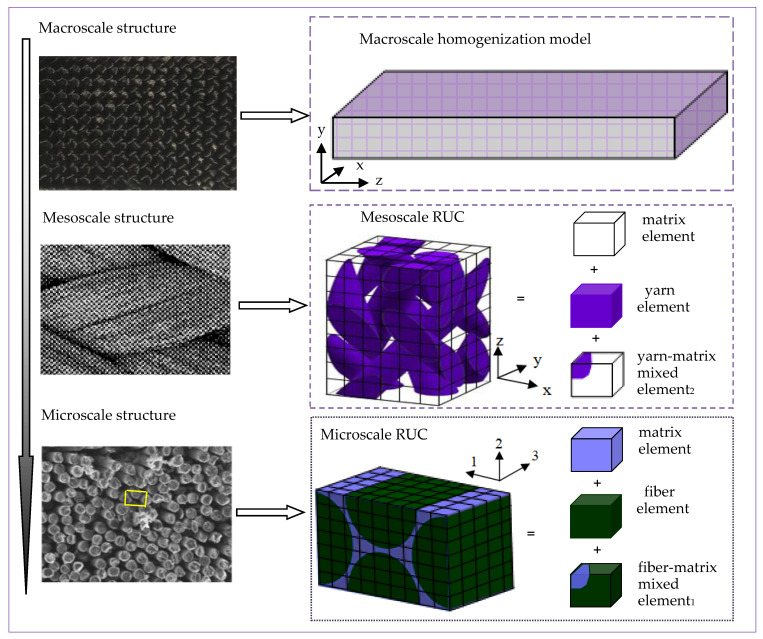
Multiscale model decomposition of 3D four-directional braided composites.

In fact, the change of material properties at a lower scale plays an important role in the failure process of macro-structure, but it is often difficult to capture the mechanical response of materials from the perspective of calculation. According to the periodical characteristic of 3D four-directional braided architecture, the numerical models and thermo-viscoelasticity behavior analysis of 3D four-directional braided composites are carried out on three-scales, such as the fiber/matrix scale, the fiber tow/matrix scale, and the homogeneous 3D braided composites scale. As shown in Figure 1, the scheme for the multi-scale modeling framework of 3D braided four-directional composites is firstly presented. The coupling technique for the multi-scale thermo-viscoelasticity behaviors prediction of 3D braided four-directional composites is given as following.

### 2.1. Association of Multiple Scales

The homogenization method is usually regarded as an effective method to solve multi-scale problems of composite materials with periodic microstructures [25,26,27], the multi-scale thermo-viscoelastic problem of 3D braided composites is solved based on the homogenization method. Since characterization of the resin-based 3D braided composites requires scale transitions between more than two scales, different models were defined. On the macro scale, the meso-scale RUC is assumed periodic. Similar to the macro-scale model, meso-scale RUC is composed of yarns and matrix, yarns are assumed to be formed from micro scale fiber bundle RUC. Let *x*, *y*, *z* denote the coordinate system of the macro-scale, the meso-scale and the micro-scale, respectively. They are related to one another scale by the positive real parameters *ε* and ω (0 < *ε* << 1, 0 < ω << 1) as follows.

(1) $$y=\frac{x}{\omega },\;z=\frac{y}{\varepsilon }.$$

### 2.2. Equations for the Micro-Scale, Meso-Scale and Macro-Scale

**Figure 2:**
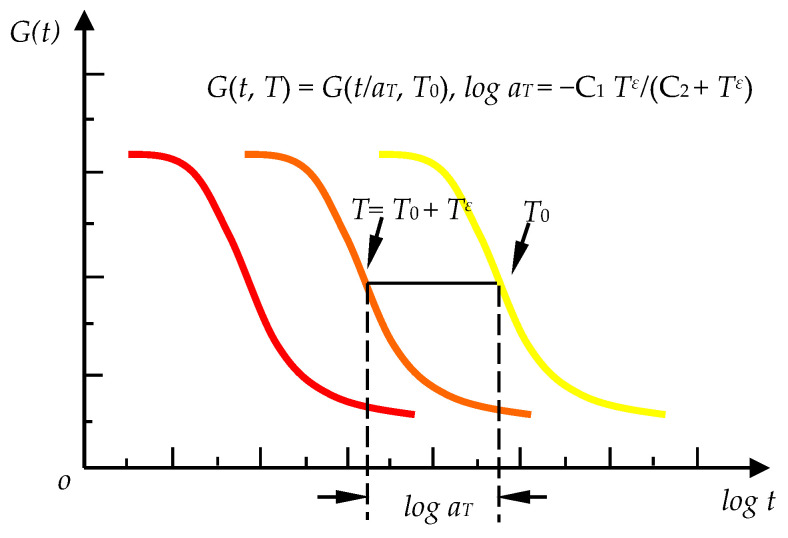
Time-temperature equivalence principle.

When the yarn of meso-scale RUC is regarded as an in-homogeneous viscoelastic medium with periodical structure, the static thermo-viscoelastic problem in equilibrium condition can be effectively expressed in *y* and *z* coordinate systems as follows [28].

(2) $$\begin{array}{l} \int_{\Omega^{\varepsilon }}\left\{\int_{0}^{t}G_{ijkl}(T,y,z,t-\tau )\frac{\partial }{\partial \tau }\left[\frac{\partial u_{k}^{\varepsilon }(y,\tau )}{\partial y_{l}}\right]d\tau -G_{ijkl}(T,y,z,t)\alpha_{kl}T^{\varepsilon }(y)\right\}\frac{\partial v_{i}}{\partial y_{j}}d\Omega \\ -\int_{\Omega^{\varepsilon }}f_{i}v_{i}d\Omega -\int_{\Gamma }t_{i}v_{i}d\Gamma =0 \end{array}$$

where $u_{k}^{\varepsilon }$ is displacement components, $\alpha_{kl}$ is thermal expansion coefficient, $T^{\varepsilon }$ is temperature increment, $f_{i}$, $t_{i}$ are body force and traction force, $v_{i}$ is virtual displacement, $\Omega^{\varepsilon }$, Γ are the yarn solid part and the boundary of domain. $G_{ijkl}$ is the static relaxation tensor at *T* of the component materials, which can be calculated by translating along the time axis based on the time-temperature equivalence principle [23], as shown in Figure 2. $\alpha_{T}$ is obtained by the Williams-Landel-Ferry equation and $C_{1}$, $C_{2}$ are the material constants.

According to the homogenization method [29], the yarn displacements in the *ε*-space are asymptotically expanded into Equation (3). The terms ${u_{i}}^{n}(n\in N_{0})$ are z-periodic functions called correctors of order *n* of the displacement field, and $u_{i}^{0}(y,t)$ is the effective displacement only depending on the meso-scopic coordinates y.

(3) $$u_{i}^{\varepsilon }(y,t)=u_{i}^{0}(y,t)+\sum_{\lambda =1}^{n}\varepsilon^{\lambda }u_{i}^{\lambda }(y,z,t)$$

The stress-strain relationship for a static thermo-viscoelastic material can be written as [28].

(4) $$\sigma_{ij}^{\varepsilon }(T,y,t)=\int_{0}^{t}G_{ijkl}(T,y,z,t-\tau )\frac{d\varepsilon_{kl}(\tau )}{d\tau }d\tau -G_{ijkl}(T,y,z,t)\alpha_{kl}T^{\varepsilon }(y)$$

The correspondence principle [30] and Laplace transformation is used to Equations (2)–(4).

(5) $$\int_{\Omega^{\varepsilon }}\left[s{\tilde{G}}_{ijkl}(T,y,z,s)\frac{\partial {\tilde{u}}_{k}^{\varepsilon }(y,s)}{\partial y_{l}}-{\tilde{G}}_{ijkl}(T,y,z,s)\alpha_{kl}T^{\varepsilon }(y)\right]\frac{\partial v_{i}}{\partial y_{j}}d\Omega -\int_{\Omega^{\varepsilon }}{\tilde{f}}_{i}v_{i}d\Omega -\int_{\Gamma }{\tilde{p}}_{i}v_{i}d\Gamma =0$$

(6) $${\tilde{\sigma }}_{ij}^{\varepsilon }(T,y,s)=s{\tilde{G}}_{ijkl}(T,y,z,s)\frac{\partial {\tilde{u}}_{k}^{\varepsilon }(y,s)}{\partial y_{l}}-{\tilde{G}}_{ijkl}(T,y,z,s)\alpha_{kl}T^{\varepsilon }(y)$$

where variables with the mark ~ show that they are Laplace transformed, *s* is the Laplace transformed parameter.

Laplace transform is used to Equation (3) and take it into Equation (5), equating the terms with the same power of ε, the following expressions can be obtained.

(7) $$\frac{1}{\varepsilon }\int_{Y_{1}}s{\tilde{G}}_{ijkl}(T,y,z,s)\left\{\left[\frac{\partial {\tilde{u}}_{k}^{0}(y,s)}{\partial y_{l}}+\frac{\partial {\tilde{u}}_{k}^{1}(y,z,s)}{\partial z_{l}}\right]-\alpha_{kl}{\tilde{T}}^{\varepsilon }(y)\right\}\frac{\partial v_{i}}{\partial z_{j}}dY_{1}=0$$

(8) $$\begin{array}{l} \varepsilon^{0}\;\int_{\Omega^{\varepsilon }}\left\{\frac{1}{\left|Y_{1}\right|}\int_{Y_{1}}s{\tilde{G}}_{ijkl}(T,y,z,s)\left[\frac{\partial {\tilde{u}}_{k}^{0}(y,s)}{\partial y_{l}}+\frac{\partial {\tilde{u}}_{k}^{1}(y,z,s)}{\partial z_{l}}-\alpha_{kl}{\tilde{T}}^{\varepsilon }(y)\right]\frac{\partial v_{i}}{\partial y_{j}}\right\}dY_{1}d\Omega \\ =\int_{\Omega^{\varepsilon }}{\tilde{f}}_{i}v_{i}d\Omega +\int_{\Gamma }{\tilde{p}}_{i}v_{i}d\Gamma \end{array}$$

As Equation (7) is linear with respect to ${\tilde{u}}_{k}^{0}$, its solution ${\tilde{u}}_{k}^{1}$ can be expressed in terms of ${\tilde{u}}_{k}^{0}$ as

(9) $${\tilde{u}}_{i}^{1}(y,z,s)=-{\tilde{\chi }}_{i}^{kl}(z,s)\frac{\partial {\tilde{u}}_{k}^{0}(y,s)}{\partial y_{l}}+{\tilde{\Phi }}_{i}(z,s){\tilde{T}}^{\varepsilon }(y),\;$$

where ${\tilde{\chi }}_{i}^{kl}$ and $\tilde{\Phi }$ represent the z-periodic characteristic displacement for viscoelasticity and thermo-viscoelasticity problem.

Replacing Equation (9) in Equation (7), the following equations can be obtained

(10) $$\int_{Y_{1}}{\tilde{G}}_{ijkl}(T,y,z,s)\frac{\partial v_{i}}{\partial z_{j}}dY_{1}-\int_{Y_{1}}{\tilde{G}}_{ijmn}(T,y,z,s)\frac{\partial {\tilde{\chi }}_{m}^{kl}(z,s)}{\partial z_{n}}\frac{\partial v_{i}}{\partial z_{j}}dY_{1}=0$$

(11) $$\int_{Y_{1}}{\tilde{G}}_{ijkl}(T,y,z,s){\tilde{\alpha }}_{kl}\frac{\partial v_{i}}{\partial z_{j}}dY_{1}-\int_{Y_{1}}{\tilde{G}}_{ijkl}(T,y,z,s)\frac{\partial {\tilde{\Phi }}_{k}(z,s)}{\partial z_{l}}\frac{\partial v_{i}}{\partial z_{j}}dY_{1}=0$$

Then, functions ${\tilde{\chi }}_{i}^{kl}$ and ${\tilde{\Phi }}_{i}$ can be obtained according to Equations (10) and (11).

According to Equation (9), Equation (4) can be written as

(12) $$\begin{array}{l} {\tilde{\sigma }}_{ij}^{0}(T,y,z,s)=s\left[{\tilde{G}}_{ijkl}(T,y,z,s)-{\tilde{G}}_{ijmn}(T,y,z,s)\frac{\partial {\tilde{\chi }}_{m}^{kl}(z,s)}{\partial z_{n}}\right]\frac{\partial {\tilde{u}}_{k}^{0}(y,s)}{\partial y_{l}}\\ -\left[{\tilde{G}}_{ijkl}(T,y,z,s)\alpha_{kl}\;-{\tilde{G}}_{ijkl}(T,y,z,s)\frac{\partial {\tilde{\Phi }}_{k}(z,s)}{\partial z_{l}}\right]{\tilde{T}}^{\varepsilon }(y) \end{array}$$

where ${\tilde{\sigma }}_{ij}^{0}(T,y,z,s)$ is the approximation of the micro-scale stress field in Laplace transformed domain.

Considering Equations (8) and (9) and taking the volume average by ${\tilde{f}}^{H}=\frac{1}{\left|Y\right|}\int_{Y}\tilde{f}dy$, the following result is obtained

(13) $$\begin{array}{l} \int_{\Omega^{\varepsilon }}\left\{\frac{1}{\left|Y_{1}\right|}\int_{Y_{1}}s\left[{\tilde{G}}_{ijkl}(T,y,z,s)-{\tilde{G}}_{ijmn}(T,y,z,s)\frac{\partial {\tilde{\chi }}_{m}^{kl}(z,s)}{\partial z_{n}}\right]\frac{\partial {\tilde{u}}_{k}^{0}(y,s)}{\partial y_{l}}dY_{1}\right\}\frac{\partial v_{i}}{\partial y_{j}}d\Omega -\\ \int_{\Omega^{\varepsilon }}\left\{\frac{1}{\left|Y_{1}\right|}\int_{Y_{1}}\left[{\tilde{G}}_{ijkl}(T,y,z,s)\alpha_{kl}-{\tilde{G}}_{ijkl}(T,y,z,s)\frac{\partial {\tilde{\Phi }}_{k}(z,s)}{\partial z_{l}}\right]dY_{1}\Delta T\right\}\frac{\partial v_{i}}{\partial y_{j}}d\Omega =\int_{\Omega^{\varepsilon }}{\tilde{f}}_{i}v_{i}d\Omega +\int_{\Gamma }{\tilde{p}}_{i}v_{i}d\Gamma \end{array}$$

Comparing with Equation (5) and according to Equations (10) and (11), the Laplace transformation of equivalent relaxation modulus of yarns ${\tilde{G}}_{ijkl}^{H1}$ and equivalent TTRM of yarns ${\tilde{\beta }}_{ij}^{H1}$ can be written as

(14) $${\tilde{G}}_{ijkl}^{H1}(T,y,s)=\frac{1}{\left|Y_{1}\right|}\int_{Y_{1}}\left[{\tilde{G}}_{ijkl}(T,y,z,s)-{\tilde{G}}_{ijmn}(T,y,z,s)\frac{\partial {\tilde{\chi }}_{m}^{kl}(z,s)}{\partial z_{n}}\right]dY_{1}$$

(15) $$\begin{array}{ll} {\tilde{\beta }}_{ij}^{H1}(T,y,s) & =\frac{1}{\left|Y_{1}\right|}\int_{Y_{1}}\left[{\tilde{G}}_{ijkl}(T,y,z,s)\alpha_{kl}-{\tilde{G}}_{ijkl}(T,y,z,s)\frac{\partial {\tilde{\Phi }}_{k}(z,s)}{\partial z_{l}}\right]dY_{1}\\ & =\frac{1}{\left|Y_{1}\right|}\int_{Y_{1}}\left[{\tilde{G}}_{ijkl}(T,y,z,s)\alpha_{kl}-{\tilde{G}}_{ijkl}(T,y,z,s)\alpha_{kl}\frac{\partial {\tilde{\chi }}_{m}^{kl}(z,s)}{\partial z_{n}}\right]dY_{1} \end{array}$$

Thus, the equivalent stress of the yarn composites in Laplace domain can be written as

(16) $${\tilde{\sigma }}_{ij}^{H1}(T,y,s)=s{\tilde{G}}_{ijkl}^{H1}(T,y,s)\frac{\partial {\tilde{u}}_{k}^{0}(y,s)}{\partial y_{l}}-{\tilde{\beta }}_{ij}^{H1}(T,y,s){\tilde{T}}^{\varepsilon }(y)$$

Under small deformation assumption and according to Liu et al. [28], the linear viscoelastic behavior of the yarn composite can be described in terms of the strain–stress relation by the Laplace inverse transformation for Equation (16).

(17) $$\sigma_{ij}^{H1}(T,y,t)=\int_{0}^{t}G_{ijkl}^{H1}(T,y,t-\tau )\frac{\partial }{\partial \tau }\frac{\partial u_{k}^{0}(y,\tau )}{\partial y_{l}}d\tau -\beta_{ij}^{H1}(T,y,t)T^{\varepsilon }(y).$$

Hence, Equation (17) can be seen as the equivalent constitutive equation of thermal stress relaxation for viscoelastic composites.

Although the constitutive equation of viscoelastic composites is similar to that of single-phase material expressed by Equation (4), the thermal stress relaxation law of the two materials is different. The thermal stress relaxation law of single-phase materials is the same as that caused by the instantaneous thermal strain $\alpha_{kl}T^{\varepsilon }$, but the thermal stress relaxation law of viscoelastic composites is different. To further illustrate, parameter $\alpha_{kl}^{H1}$ is introduced and the equivalent TTRM ${\tilde{\beta }}_{ij}^{H1}$ can be written as

(18) $${\tilde{\beta }}_{ij}^{H1}(T,y,t)={\tilde{G}}_{ijkl}^{H1}(T,y,t)\;\alpha_{kl}^{H1}$$

Therefore, the following form of parameter $\alpha_{kl}^{H1}$ can be obtained and it can be found that the parameter $\alpha_{kl}^{H1}$ is time-dependent. At the same time, it can also be known that the strain caused by initial temperature increment has a development process, which cannot be completed instantaneously for viscoelastic composites.

(19) $$\alpha_{kl}^{H1}(t)={\left[G_{ijkl}^{H1}(T,y,t)\right]}^{-1}\beta_{ij}^{H1}(T,y,t)$$

Replacing Equation (17) by Equation (19), the following result is given and the parameter $\alpha_{kl}^{H1}(t)$ can be defined as equivalent TTEC by comparing with Equation (4).

(20) $$\sigma_{ij}^{H1}(T,y,t)=\int_{0}^{t}G_{ijkl}^{H1}(T,y,t-\tau )\frac{\partial }{\partial \tau }\frac{\partial u_{k}^{0}(y,\tau )}{\partial y_{l}}d\tau -G_{ijkl}^{H1}(T,y,t)\alpha_{kl}^{H1}(t)T^{\varepsilon }(y)$$

When the 3D braided composites of macro-scale is regarded as an in-homogeneous viscoelastic medium with periodical meso-scale RUC, the static thermo-viscoelastic problem in equilibrium condition can be effectively expressed in x and y coordinate systems as follows.

(21) $$\begin{array}{l} \int_{{\overset{⌢}{\Omega }}^{\omega }}\left\{\int_{0}^{t}G_{ijkl}(T,x,y,t-\tau )\frac{\partial }{\partial \tau }\left[\frac{\partial u_{k}^{\omega }(x,\tau )}{\partial x_{l}}\right]d\tau -G_{ijkl}(T,x,y,t)\alpha_{kl}(t)T^{\varepsilon }(x)\right\}\frac{\partial v_{i}}{\partial x_{j}}d\overset{⌢}{\Omega }\\ =\int_{{\overset{⌢}{\Omega }}^{\omega }}{\overset{⌢}{f}}_{i}v_{i}d\overset{⌢}{\Omega }+\int_{\Gamma }{\overset{⌢}{p}}_{i}v_{i}d\overset{⌢}{\Gamma } \end{array}$$

where ${\overset{⌢}{f}}_{i}$ is body force, ${\overset{⌢}{p}}_{i}$, $v_{i}$ are traction force and virtual displacement, $G_{ijkl}$ is the static relaxation tensor of the component materials. $u_{k}^{\omega }$ is the displacement, $\alpha_{kl}(t)$ is the time-dependent thermal expansion coefficient, $\overset{⌢}{\Omega }{\;}^{\omega }$, $\overset{⌢}{\Gamma }$ are the macro-scale solid part and the boundary of the domain.

Based on the homogenization method, a perturbation of the macro-scale displacement vector $u_{i}^{0}(x,t)$ is performed with respect to the meso-scale coordinate system y.

(22) $$u_{i}^{\omega }(x,t)=u_{i}^{0}(x,t)+\omega u_{i}^{1}(x,y,t)+\omega_{i}^{2}u_{i}^{2}(x,y,t)+\ldots$$

Applying the correspondence principle to thermo-viscoelastic analysis of macro-scale and meso-scale, Laplace transform is made to Equations (21) and (22). By substituting Equation (22) into Equation (21) and sorting out the items about ω. Considering the coefficient of $\omega^{-1}$ and $\omega^{0}$, the following equations can be obtained according to the homogenization theory [28].

(23) $$\int_{Y_{2}}s{\tilde{G}}_{ijkl}(T,x,y,s)\left\{\left[\frac{\partial {\tilde{u}}_{k}^{0}(x,s)}{\partial x_{l}}+\frac{\partial {\tilde{u}}_{k}^{1}(x,y,s)}{\partial y_{l}}\right]-{\tilde{\alpha }}_{kl}(s){\tilde{T}}^{\varepsilon }(x)\right\}\frac{\partial v_{i}}{\partial z_{j}}dY_{2}=0$$

(24) $$\begin{array}{l} \;\int_{{\overset{⌢}{\Omega }}^{\omega }}\left\{\frac{1}{\left|Y_{2}\right|}\int_{Y_{2}}s{\tilde{G}}_{ijkl}(T,x,y,s)\left[\frac{\partial {\tilde{u}}_{k}^{0}(x,s)}{\partial x_{l}}+\frac{\partial {\tilde{u}}_{k}^{1}(x,y,s)}{\partial y_{l}}-{\tilde{\alpha }}_{kl}(s){\tilde{T}}^{\varepsilon }(x)\right]\frac{\partial v_{i}}{\partial y_{j}}\right\}dY_{2}d\overset{⌢}{\Omega }\\ =\int_{{\overset{⌢}{\Omega }}^{\omega }}{\tilde{\overset{⌢}{f}}}_{i}v_{i}d\overset{⌢}{\Omega }+\int_{\Gamma }{\tilde{\overset{⌢}{p}}}_{i}v_{i}d\overset{⌢}{\Gamma } \end{array}$$

The separation variable method is used to solve Equation (23), ${\tilde{u}}_{k}^{1}(x,y,s)$ can be expressed in terms of ${\tilde{u}}_{k}^{0}(x,s)$ as

(25) $${\tilde{u}}_{i}^{1}(x,y,s)=-{\tilde{X}}_{i}^{kl}(y,s)\frac{\partial {\tilde{u}}_{k}^{0}(x,s)}{\partial x_{l}}+{\tilde{\Psi }}_{i}(y,s){\tilde{T}}^{\varepsilon }(x)$$

where ${\tilde{Χ}}_{m}^{kl}$ and ${\tilde{\Psi }}_{k}$ represent the y-periodic characteristic displacement for viscoelasticity and thermo-viscoelasticity problem, respectively.

Replacing Equation (25) by Equation (23), the characteristic displacement field vector ${\tilde{Χ}}_{m}^{kl}$ and ${\tilde{\Psi }}_{k}$ are the solution of the following equations:

(26) $$\int_{Y_{2}}\left[{\tilde{G}}_{ijkl}(T,x,y,s)-{\tilde{G}}_{ijmn}(T,x,y,s)\frac{\partial {\tilde{Χ}}_{m}^{kl}(y,s)}{\partial y_{n}}\right]\frac{\partial v_{i}}{\partial y_{j}}dY_{2}=0$$

(27) $$\int_{Y_{2}}\left[{\tilde{G}}_{ijkl}(T,x,y,s)\frac{\partial {\tilde{\Psi }}_{k}(y,s)}{\partial y_{l}}-{\tilde{G}}_{ijkl}(T,x,y,s){\tilde{\alpha }}_{kl}(s)\right]\frac{\partial v_{i}}{\partial y_{j}}dY_{2}=0$$

Replacing Equation (24) in Equation (25), the Laplace transformation of equivalent relaxation modulus ${\tilde{G}}_{ijkl}^{H2}$, equivalent TTRM ${\tilde{\beta }}_{ij}^{H2}$ and equivalent TTEC ${\tilde{\alpha }}_{ij}^{H2}$ for the meso-scale RUC can be similarly expressed as:

(28) $${\tilde{G}}_{ijkl}^{H2}(T,x,s)=\frac{1}{\left|Y_{2}\right|}\int_{Y_{2}}\left[{\tilde{G}}_{ijkl}(T,x,y,s)-{\tilde{G}}_{ijmn}(T,x,y,s)\frac{\partial {\tilde{Χ}}_{m}^{kl}(y,s)}{\partial y_{n}}\right]dY_{2}$$

(29) $$\begin{array}{ll} {\tilde{\beta }}_{ij}^{H2}(T,x,s) & =\frac{1}{\left|Y_{2}\right|}\int_{Y_{2}}\left[{\tilde{G}}_{ijkl}(T,x,y,s){\tilde{\alpha }}_{kl}(s)-{\tilde{G}}_{ijkl}(T,x,y,s)\frac{\partial {\tilde{\Psi }}_{k}(y,s)}{\partial y_{l}}\right]dY_{2}\\ & =\frac{1}{\left|Y_{2}\right|}\int_{Y_{2}}\left[{\tilde{G}}_{ijkl}(T,x,y,s){\tilde{\alpha }}_{kl}(s)-{\tilde{G}}_{ijmn}(T,x,y,s){\tilde{\alpha }}_{kl}(s)\frac{\partial {\tilde{Χ}}_{m}^{kl}(y,s)}{\partial y_{n}}\right]dY_{2} \end{array}$$

(30) $${\tilde{\alpha }}_{ij}^{H2}(s)={\left[{\tilde{G}}_{ijkl}^{H2}(T,x,s)\right]}^{-1}{\tilde{\beta }}_{kl}^{H2}(T,x,s)$$

According to Equations (21) and (22), the approximation of the meso-scale stress field in Laplace transformed domain can be written as

(31) $$\begin{array}{l} {\tilde{\sigma }}_{ij}^{0}(T,x,y,s)=s\left[{\tilde{G}}_{ijkl}(T,x,y,s)-{\tilde{G}}_{ijmn}(T,x,y,s)\frac{\partial {\tilde{Χ}}_{m}^{kl}(y,s)}{\partial y_{n}}\right]\frac{\partial {\tilde{u}}_{k}^{0}(x,s)}{\partial x_{l}}\\ -\left[{\tilde{G}}_{ijkl}(T,x,y,s){\tilde{\alpha }}_{kl}(s)-{\tilde{G}}_{ijkl}(T,x,y,s)\frac{\partial {\tilde{\Psi }}_{k}(y,s)}{\partial y_{l}}\right]{\tilde{T}}^{\varepsilon }(x) \end{array}$$

Based upon the above derivation, the macroscopic equivalent stress vector ${\tilde{\sigma }}_{ij}^{H2}$ can be expressed as

(32) $${\tilde{\sigma }}_{ij}^{H2}(T,x,s)=s{\tilde{G}}_{ijkl}^{H2}(T,x,s)\frac{\partial {\tilde{u}}_{k}^{0}(x,s)}{\partial x_{l}}-{\tilde{\beta }}_{ij}^{H2}(T,x,s){\tilde{T}}^{\varepsilon }(x)$$

## 3. Analysis Procedure for 3D Braided Composites

### 3.1. Inverse Laplace Transformation

Based on the previous derivation, the Laplace transform of homogenized viscoelastic behavior is solved by using the commonly used homogenization technique of elastic materials in the transformation space. In order to obtain this result, the inverse Laplace transform is needed to calculated the homogenized thermal-viscoelastic behavior in the time domain.

Assuming that the viscoelastic properties of 3D braided composites satisfy the following three-parameter model [31], the viscoelastic functions can be represented by the n-order Prony series expressed as follows

(33) $$G(t)=G_{\infty }+\sum_{i=1}^{n}G_{i}e^{-\frac{t}{\tau_{i}}},$$

where the $G_{\infty }$, $G_{i}$, $\tau_{i}$ are the long-term modulus, Prony coefficients and relaxation times, respectively.

In the Laplace transformed domain, the viscoelastic functions can be expressed as

(34) G˜(s)=G∞s+∑i=1nGis+1τi,

where the non-linear least square method [32] is used to calculate the coefficients $G_{\infty }$, $G_{i}$, $\tau_{i}$. Then, the equivalent thermo-relaxation response in the time domain can be considered as Equation (33).

### 3.2. Computation of the Effective Properties for Micro-Scale and Meso-Scale

In order to analyze the thermo-viscoelastic behavior of 3D braided composites, the details of the FE method for computing the micro-scale characteristic functions ${\tilde{\chi }}_{i}^{kl}$ and meso-scale characteristic function ${\tilde{Χ}}_{i}^{kl}$ are necessary.

The FE discretized form of Equations (10) and (26) would be written as

(35) $$\left[\begin{array}{cc} \left[{\tilde{K}}^{z}\right] & \left[0\right]\\ \left[0\right] & \left[{\tilde{K}}^{y}\right] \end{array}\right]\;\left\{\begin{array}{c} \left[\tilde{\phi }\right]\\ \left[\tilde{\vartheta }\right] \end{array}\right\}=\left\{\begin{array}{c} \left[F^{z}\right]\\ \left[F^{y}\right] \end{array}\right\}$$

where

(36) $${\tilde{K}}^{z}={\int_{Z}B^{T}\tilde{G}}^{z}BdZ$$

(37) $${\tilde{K}}^{y}={\int_{Y}B^{T}\tilde{G}}^{y}BdY$$

(38) $${\tilde{F}}^{z}=\int_{Z}B^{T}{\tilde{G}}^{z}dZ$$

(39) $${\tilde{F}}^{y}=\int_{Y}B^{T}{\tilde{G}}^{y}dY,$$

where *B* is the shape function derivative matrix. ${\tilde{G}}^{q}\left(q=z,y\right)$ is the Laplace transformed relaxation tensor matrix of the micro or meso-scale [13]. $\tilde{\phi }$ and $\tilde{\vartheta }$ are the discretized form of the corrector $\tilde{\chi }$ and $\tilde{Χ}$. The right term of Equation (35) is a matrix ${\tilde{F}}^{q}\left(q=z,y\right)$.

To obtain the matrix $\tilde{\phi }$ and $\tilde{\vartheta }$ in Equation (35), the multi-phase finite element (MPFE) method [33] was employed. A micro-scale RUC and a meso-scale RUC are composed of six kinds of 20-node rectangular isoparametric elements in Figure 1. Based on the MPFE method, the Gauss quadrature formulae are employed to calculate all element integrals in Equations (36)–(39). In addition, 27 Gauss integral points are selected in each kind of element in this work.

(40) $${\tilde{K}}^{L}=\sum_{n=1}^{N}\left(\sum_{ii=1}^{3}\sum_{jj=1}^{3}\sum_{kk=1}^{3}W_{ii}{\bar{W}}_{jj}{\bar{\bar{W}}}_{kk}{\left({\left[B\right]}^{T}\left[{\tilde{G}}_{\alpha }\right]\left[B\right]\det\left[J\right]\right)}_{\xi =\xi_{ii},\;\eta =\eta_{jj},\;\zeta =\zeta_{kk}}\right),L=z,y$$

(41) $${\tilde{F}}^{L}=\sum_{n=1}^{N}\left(\sum_{ii=1}^{3}\sum_{jj=1}^{3}\sum_{kk=1}^{3}W_{ii}{\bar{W}}_{jj}{\bar{\bar{W}}}_{kk}{\left({\left[B\right]}^{T}\left[{\tilde{G}}_{\alpha }\right]\det\left[J\right]\right)}_{\xi =\xi_{ii},\;\eta =\eta_{jj},\;\zeta =\zeta_{kk}}\right),\alpha =fi,ma,mix_{1},mix_{2}$$

where *N* is the number of 20-node rectangular isoparametric elements of micro-scale RUC or meso-scale RUC, respectively. ${\tilde{G}}_{\alpha }$ is the material property matrix of components, $W_{ii},{\bar{W}}_{jj},{\bar{\bar{W}}}_{kk}$ is the corresponding weights. J is the Jacobian matrix.

On the premise of fine mesh generation and convergence of finite element results, the matrix $\tilde{\phi }$ and the homogenized effective thermo-viscoelastic properties of the yarns can be obtained firstly. Similarly, once the matrix $\tilde{\vartheta }$ has been determined, the homogenized effective thermo-viscoelastic properties of the meso-scale RUC can be obtained.

### 3.3. FsMsFE Analysis Sequence

In the following, a detailed description of the proposed five-step multi-scale strategy for determining the thermo-viscoelastic behaviors of 3D braided composites will be given.

(1) Establish a micro-scale RUC model. Then, Equation (35) is solved to obtain the characteristic function $\tilde{\phi }$. Thereafter, the equivalent thermo-viscoelastic properties ${\tilde{\alpha }}_{kl}^{H1}$ and ${\tilde{\beta }}_{ij}^{H1}$ of the yarns are computed by Equations (15) and (19).
(2) Establish a meso-scale RUC model of 3D four-directional braided composites. The characteristic function $\tilde{\vartheta }$ for the meso-scale unit cell of 3D braided four-directional composites can be obtained similar to that of micro-scale RUC. Then the equivalent TTRM ${\tilde{\beta }}_{ij}^{H2}$ and equivalent TTEC ${\tilde{\alpha }}_{ij}^{H2}$ of the meso-scale RUC can be calculated by means of Equations (29) and (30).
(3) Establish a macroscopic model with uniform mechanical properties, which was calculated from homogenization analysis of meso-scale RUC. Based on a certain boundary condition, the homogenization displacement ${\tilde{u}}_{k}^{0}(x,s)$ can be obtained.
(4) Calculate the meso-scale stress field inside the basic RUC belonging to the macroscopic location by Equation (31).
(5) Calculate the micro-scale stress field related to the yarn by Equation (12).

## 4. Results and Discussion

### 4.1. Numerical Models and Verification

**Figure 3:**
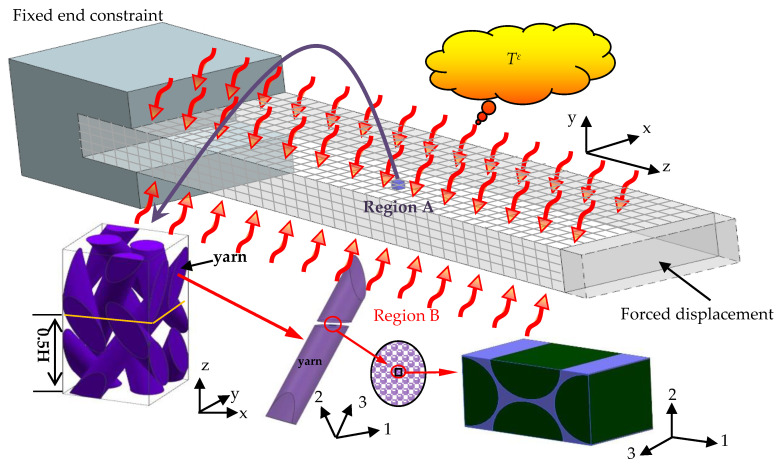
Sketch of a beam under thermo-mechanical coupling.

In this section, the thermo-viscoelastic behaviors of 3D four-directional braided composites are studied based on the FsMsFE method presented in this paper. The reinforced fiber is T300 carbon and the matrix is epoxy resin ED-6, respectively. The material parameters of the carbon fiber and resin are shown in Table 1. In this work, the time-dependent thermo-viscoelastic response of the 3D braided composite clamped-free beam with an instantaneous temperature increment $T^{\varepsilon }$ and a certain forced displacement at the free edge are studied. As shown in Figure 3, the dimension of the beam is 90 × 20 × 6 mm^3^, the temperature increment is $T^{\varepsilon }$ and the forced displacement at the free edge is *d* = 0.1 mm.

When the fiber volume fraction of the yarn is 0.28, the microscale RUC consisted of 3024 20-node rectangular isoparametric elements is used to calculate the effective relaxation modulus of the yarn and verify the accuracy of the present FsMsFE method. To obtain the effective relaxation modulus of the yarn by Equation (14), it is necessary to calculate the corrector $\tilde{\chi }$ via Equation (35). In this work, it is assumed that the reinforcement phase is considered as elastic transversely isotropic material. The volume deformation of the matrix is considered to be linear elastic and the shear deformation conforms to the three-parameter solid model. Therefore, the material property matrix *G_fi_* and *G_ma_* for reinforcement fiber and matrix can be written as

(42) $$\left[G_{fi}\right]={\left[\begin{array}{cccccc} \frac{1}{E_{1}} & -\frac{\gamma_{21}}{E_{1}} & -\frac{\gamma_{13}}{E_{3}} & 0 & 0 & 0\\ -\frac{\gamma_{21}}{E_{1}} & \frac{1}{E_{1}} & -\frac{\gamma_{13}}{E_{3}} & 0 & 0 & 0\\ -\frac{\gamma_{13}}{E_{3}} & -\frac{\gamma_{13}}{E_{3}} & \frac{1}{E_{3}} & 0 & 0 & 0\\ 0 & 0 & 0 & \frac{1}{G_{12}} & 0 & 0\\ 0 & 0 & 0 & 0 & \frac{1}{G_{13}} & 0\\ 0 & 0 & 0 & 0 & 0 & \frac{1}{G_{13}} \end{array}\right]}^{-1}$$

(43) $$\left[G_{ma}\right]=\left[\begin{array}{cccccc} \bar{K}+\frac{4}{3}Y(t) & \bar{K}-\frac{2}{3}Y(t) & \bar{K}-\frac{2}{3}Y(t) & 0 & 0 & 0\\ \bar{K}-\frac{2}{3}Y(t) & \bar{K}+\frac{4}{3}Y(t) & \bar{K}-\frac{2}{3}Y(t) & 0 & 0 & 0\\ \bar{K}-\frac{2}{3}Y(t) & \bar{K}-\frac{2}{3}Y(t) & \bar{K}+\frac{4}{3}Y(t) & 0 & 0 & 0\\ 0 & 0 & 0 & Y(t) & 0 & 0\\ 0 & 0 & 0 & 0 & Y(t) & 0\\ 0 & 0 & 0 & 0 & 0 & Y(t) \end{array}\right]$$

(44) $$Y(t)=2\left(\frac{G_{1}G_{1}}{G_{1}+G_{2}}+\frac{G_{1}^{2}}{G_{1}+G_{2}}e^{-t/(\frac{\eta_{2}}{G_{1}+G_{2}})}\right),$$

where $\bar{K}$, Y(t) are volume modulus and shear modulus, respectively. $G_{1}$, $G_{2}$, *η*_2_ are the viscoelastic parameters of matrix. $E_{1}$, $E_{3}$, $\gamma_{21}$, $\gamma_{13}$, $G_{12}$ and $G_{13}$ are the elastic parameters of reinforcement phase.

**Figure 4:**
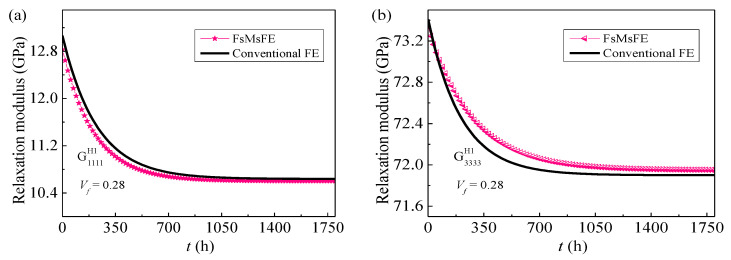
Comparison of the homogenized effective relaxation modulus of yarns calculated via five-step multi-scale finite element (FsMsFE) method with conventional FE method transverse (**a**) the transverse $G_{1111}^{H1}$; (**b**) the longitudinal $G_{3333}^{H1}$.

In fof a beam under thermo-mec [34] are put forward to solve the micro-scale problem. When the corrector $\tilde{\chi }$ based on the microscale RUC is obtained, Figure 4 shows the comparison between equivalent viscoelastic properties of the yarns obtained by FsMsFE method and that evaluated by conventional FE method [35]. It is found that the present results are in good agreement with the existing data in the literature and the differences caused by different discretization methods is kept in a small range.

### 4.2. Effective Thermo-Viscoelastic Properties

**Figure 5:**
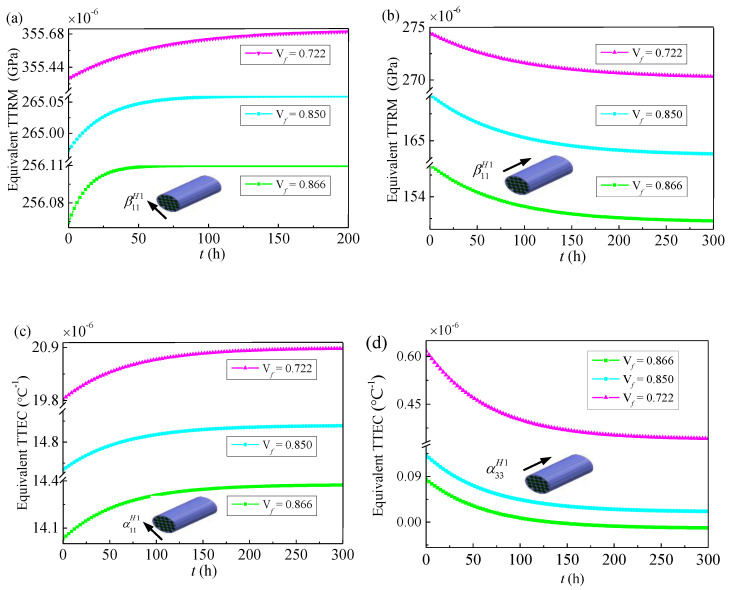
Equivalent time-dependent thermal relaxation modulus and equivalent time-dependent thermal expansion coefficients of yarns versus time for different fiber volume content (ground state) (**a**) the transverse TTRM $\beta_{11}^{H1}$; (**b**) the longitudinal TTRM $\beta_{33}^{H1}$; (**c**) the transverse TTEC $\alpha_{11}^{H1}$; (**d**) the longitudinal TTEC $\alpha_{33}^{H1}$.

**Figure 6:**
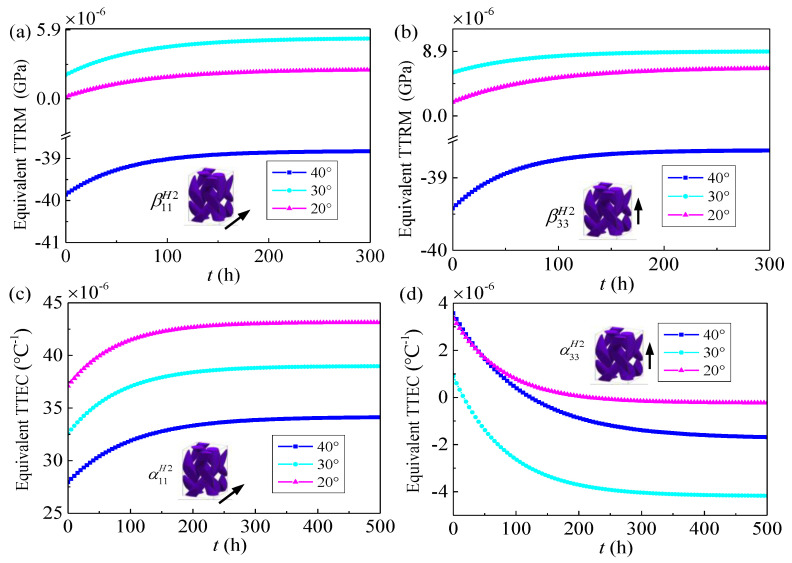
Equivalent time-dependent thermal relaxation modulus and equivalent time-dependent thermal expansion coefficients of mesoscale RUC2 versus time for different braiding angles (Ground state) (**a**) the transverse TTRM $\beta_{11}^{H2}$; (**b**) the longitudinal TTRM $\beta_{33}^{H2}$; (**c**) the transverse TTEC $\alpha_{11}^{H2}$; (**d**) the longitudinal TTEC $\alpha_{33}^{H2}$.

As mentioned in Section 2, to obtain thermo-viscoelastic response of 3D four-directional braided composites, the effective thermo-viscoelastic properties of the yarn and meso-scale RUC should be firstly evaluated. Owing to the thermo-viscoelastic properties for composites are temperature dependent, the effective TTRM $\beta_{ij}^{H1}$, $\beta_{ij}^{H2}$ and effective TTEC $\alpha_{kl}^{H1}$, $\alpha_{11}^{H2}$ of different scales at room temperature 25 °C (ground state) are presented in Figure 5 and Figure 6.

In Figure 5a,b, the yarns are considering the unidirectional reinforced composite with a constant fiber volume fraction 0.722, 0.850 and 0.866, the transverse equivalent TTRM $\beta_{11}^{H1}$ increases monotonically while the longitudinal equivalent TTRM $\beta_{33}^{H1}$ decreases monotonically with the increasing of the time, the transverse equivalent TTRM $\beta_{11}^{H1}$ and longitudinal equivalent TTRM $\beta_{33}^{H1}$ show a downward trend with increasing fiber volume fraction. A similar scenario of effective thermal expansion coefficients $\alpha_{kl}^{H1}$ in both transverse and longitudinal is shown in Figure 5c,d and they will be reached a steady state. As shown in Figure 6, the effective thermo-viscoelastic properties of the meso-scale RUC under different braiding angles (20°, 30°, 40°) and fiber volume fraction 0.37 are calculated. It can be seen that both the transverse and longitudinal equivalent TTRM $\beta_{ij}^{H2}$ are firstly decrease and then increase when the braiding angle increases and the transverse effective TTEC $\alpha_{11}^{H2}$ decrease monotonically with the increasing of the braiding angle when the longitudinal effective TTEC $\alpha_{33}^{H2}$ firstly increase and then decrease. Additionally, Figure 5 and Figure 6 show that the different fiber fraction and braiding angle in the ground state will affect the thermo-viscoelastic properties of composite in time domain.

**Figure 7:**
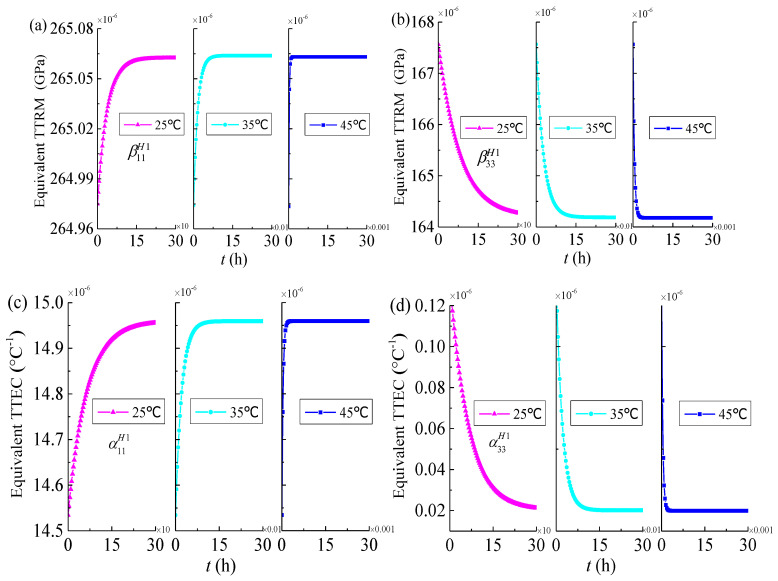
Equivalent time-dependent thermal relaxation modulus and equivalent time-dependent thermal expansion coefficients of yarns versus time for different temperatures (*V_f_* = 0.85) (**a**) the transverse TTRM $\beta_{11}^{H1}$; (**b**) the longitudinal TTRM $\beta_{33}^{H1}$; (**c**) the transverse TTEC $\alpha_{11}^{H1}$; (**d**) the longitudinal TTEC $\alpha_{33}^{H1}$.

**Figure 8:**
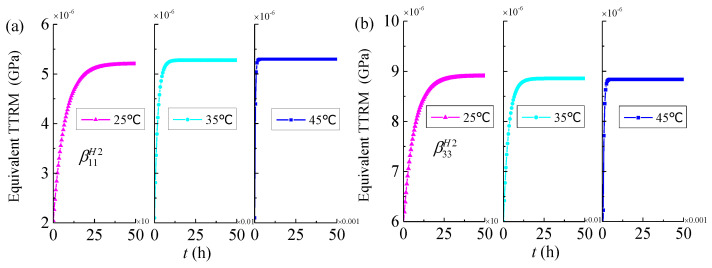

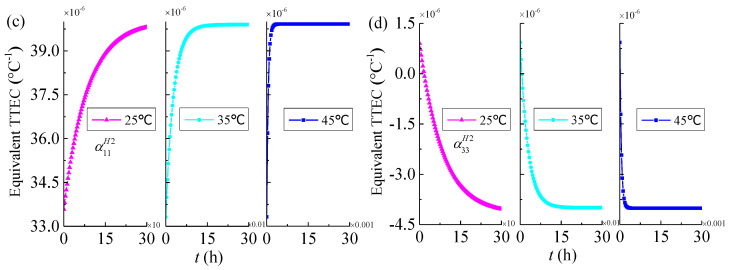
Equivalent time-dependent thermal relaxation modulus and equivalent time-dependent thermal expansion coefficients of mesoscale RUC_2_ versus time for different temperatures (*V_f_* = 0.37, φ = 30°) (**a**) the transverse TTRM $\beta_{11}^{H2}$; (**b**) the longitudinal TTRM $\beta_{33}^{H2}$; (**c**) the transverse TTEC $\alpha_{11}^{H2}$; (**d**) the longitudinal TTEC $\alpha_{33}^{H2}$.

In order to observe the effects of temperature on the thermo-viscoelastic properties of 3D four directional braided composites, the analysis results of yarns (*V_f_* = 0.85) and meso-scale RUC_2_ (*V_f_* = 0.37, φ = 30°) at different temperatures (25 °C, 35 °C, 45 °C) are shown in Figure 7 and Figure 8. It can be found that the influence of temperature on thermo-viscoelastic properties of 3D four directional braided composites is very obvious and higher the temperature the shorter the time required for the effective TTRM and effective TTEC to stabilize. This phenomenon is similar to the effect of temperature on the viscoelastic properties of 3D composites reported by Seifert et al. [21].

### 4.3. Time-Dependent Multiscale Stress Field Distributions

The initial damage of 3D braided composites is commonly attributed to the destruction of different scales, therefore a multiscale stress relaxation analysis is given in this section. Based on the different thermal and mechanical loading boundary conditions, the macro/meso/micro-scale time-dependent stresses can be obtained by using the FsMsFE analysis method presented in the Section 2.2 and Section 3.1.

**Figure 9:**
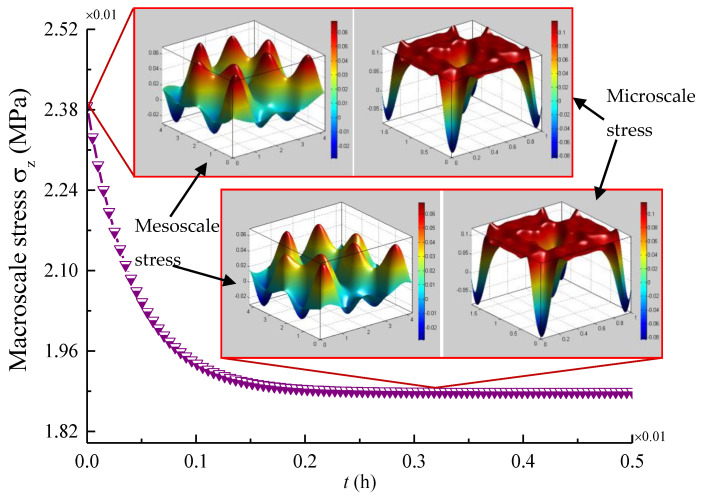
Multi-scale stress $\sigma_{z}$ of a 3D braided composite beam in time domain at 45 °C.

When the braiding parameters are determined (*V_f_* = 0.37, φ = 30°) and the temperature is 45 °C, Figure 9 presents the macro-scale stress $\sigma_{z}$ of position A, the meso-scale stress $\sigma_{z}$ on the plane (Z = 0.5H) of a RUC and the micro-scale stress $\sigma_{3}$ (fiber direction) belong to the region B of the mesoscopic yarn. It can be noted that the stress relaxation response of 3D four-directional braided composites consists of a shorter relaxation stage and a longer steady stage. At the same moment, the smaller the selected scale the greater the stresses.

**Figure 10:**
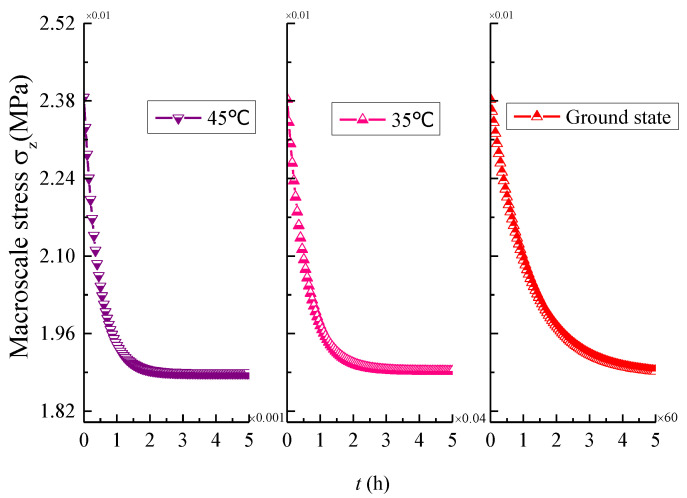
Comparison of the macro-scale stress $\sigma_{z}$ under different temperatures.

**Figure 11:**
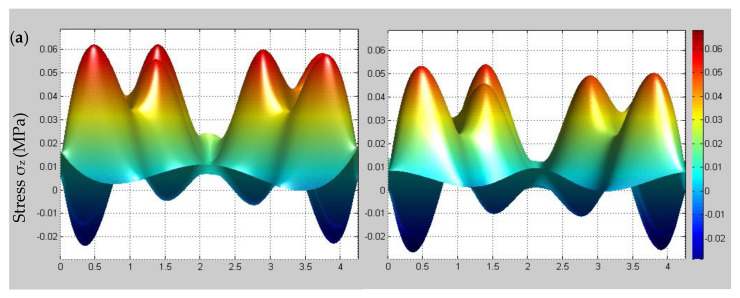

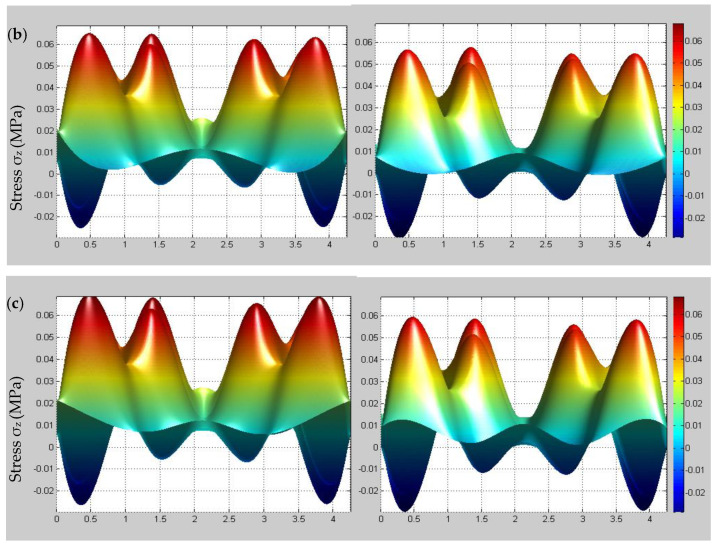
Comparison of the meso-scale stress $\sigma_{z}$ before and after stabilization on the plane Z = H/2 for a unit cell at different temperatures. (**a**) Ground state; (**b**) 35 °C; (**c**) 45 °C.

**Figure 12:**
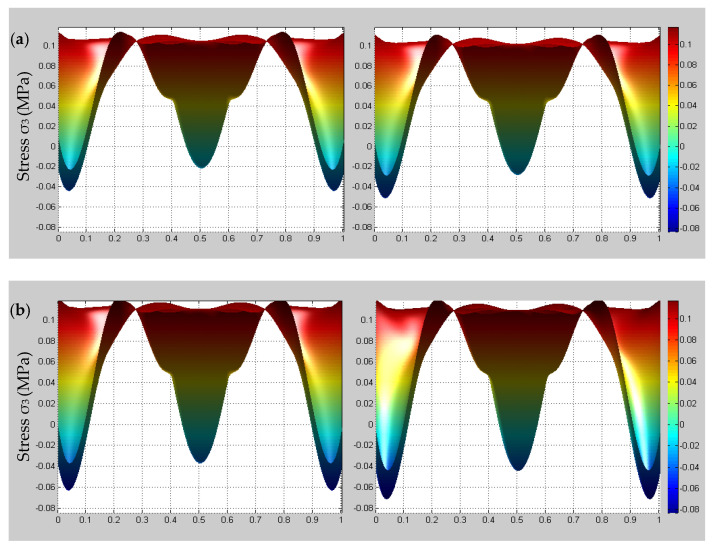

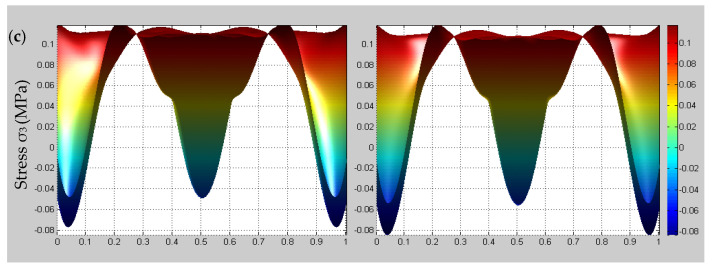
Comparison of the micro-scale stress $\sigma_{3}$ for a yarn before and after stabilization at different temperatures. (**a**) Ground state; (**b**) 35 °C; (**c**) 45 °C.

In order to determine the influence of temperature changes on the multi-scale stress of 3D braided composites, the initial and stable state stress distributions of different scales at different temperatures (25 °C, 35 °C, 45 °C) are presented in Figure 10, Figure 11 and Figure 12.

In Figure 10, it is shown that the macroscopic stress peak value change is small, the temperature rise causes the macroscopic stress stabilization time to be significantly reduced. Simultaneously, it can be observed that the time-dependent stress under the fixed displacement boundary condition at ground state is consistent with the trend described by Tate et al. [16]. From Figure 11, it is found that both meso-scale stress increases significantly before and after stabilization under the influence of temperature changes and the tensile load is mainly shared by the braided yarns. As shown in Figure 12, it can be observed that there is a more obvious micro-scale stress change for matrix than the fiber region under the influence of temperature changes. Based on the above phenomenon, it can be noted that the initial damage probability of 3D braided composites will increase and the viscoelastic effect in the micro-scale fiber/matrix model cannot be neglected when the temperature increment is considered.

## 5. Conclusions

This article presents a novel FsMsFE calculation procedure for simulating the thermo-viscoelastic response of 3D braided four-directional composites. The integration of FE calculation programs is more flexible and the dependence on the microstructure type is less through the developed Fortran calculation procedure. The five-step homogenization is carried out at micro-scale, meso-scale and macro-scale and the equivalent thermo-viscoelastic properties and stress relaxation at three scales for 3D braided composite subject to temperature increment and forced displacement are investigated, respectively. The thermo-viscoelastic properties of the 3D braided composites are obviously anisotropic and time-temperature-dependent. The stress relaxation behavior of 3D braided composites is greatly affected by temperature and temperature increment will shorten the relaxation stage of 3D braided composites and aggravate the material failure. It shows obvious non-uniformity for the stress distribution of micro-scale RUC and mesoscale RUC, especially at the interfaces of the fiber/matrix or yarn/matrix. This method can be used for the structural design of braided composite materials when the viscoelasticity and temperature rise of the material are considered.

## Figures and Tables

**Table 1:** Component material parameters of 3D braided composites in room temperature [23].

Phase	Parameters	Value
Reinforced fiber	Transverse modulus *E_f_*_1_ (GPa)	13.8
	Longitudinal modulus *E_f_*_3_ (GPa)	220
	Shear modulus *G_f_*_13_ (GPa)	9.0
	Shear modulus *G_f_*_12_ (GPa)	5.52
	Longitudinal poisson’s ratio *γ_f_*_12_	0.25
	Transverse poisson’s ratio *γ_f_*_31_	0.3
	Longitudinal thermal expansion coefficient *α*_11_ (10^−6^/K)	−0.3
	Transverse thermal expansion coefficient *α*_22_ (10^−6^/K)	8.0
Matrix	Elastic coefficient *G*_1_ (GPa)	3.2
	Elastic coefficient *G*_2_ (GPa)	1.8
	Viscosity coefficient *η*_2_ (GPa·h)	300
	Volume modulus *K* (GPa)	5.56
	Thermal expansion coefficient *α_m_* (10^−6^/K)	54

## Data Availability

Data sharing is not applicable to this article.

## References

[1] Sun J., Zhou G., Zhou C. (2015). Microstructure and mechanical properties of 3D surface-core 4-directional braided composites. J. Mater. Sci..

[2] Zhang W., Ding X., Li Y. (2008). Calculation and design of parameters for four-step 3D braided preform with complex rectangular cross sections. J. Ind. Text..

[3] Xu K., Qian X. (2015). Microstructure analysis and multi-unit cell model of three dimensionally four-directional braided composites. Appl. Compos. Mater..

[4] Zhang C., Curiel-Sosa J.L., Duodu E.A. (2017). Finite element analysis of the damage mechanism of 3D braided composites under high-velocity impact. J. Mater. Sci..

[5] Zhao Z., Dang H., Xing J. (2019). Progressive failure simulation of notched tensile specimen for triaxially-braided composites. Materials.

[6] Zuo H., Li D., Jiang L. (2019). High Temperature Mechanical Response and Failure Analysis of 3D Five-Directional Braided Composites with Different Braiding Angles. Materials.

[7] Liu J., Chen L., Li D., Li J. (2004). Analysis of creep behavior of resin-based 3-D braided composites. J. Tianjin Poly. Univ..

[8] Li D., Li J., Chen L., Lu Z. (2006). Studies on creep behavior of three dimensional braiding composites. J. Aeronaut. Mater..

[9] Priyank U., Upadhyay C. (2011). A three-dimensional micromechanical model to predict the viscoelastic behavior of woven composites. Compos. Struct..

[10] Yuan X., Sun H. (2012). Viscoelastic properties of 3D 4-directional braided composites. Acta. Mater. Compos. Sin..

[11] Cai Y., Sun H. (2013). Prediction on viscoelastic properties of three-dimensionally braided composites by multi-scale model. J. Mater. Sci..

[12] Mourid A., Ganesan R., Levesque M. (2013). Comparison between analytical and numerical predictions for the linearly viscoelastic behavior of textile composites. Mech. Mater..

[13] Zhou C., Zhang Y. (2007). Multiple scale viscoelastic analysis of 3D woven composite materials. Acta Mater. Compos. Sin..

[14] Liu X., Tang T., Yu W., Pipes R. (2018). Multiscale modeling of viscoelastic behaviors of textile composites. Int. J. Eng. Sci..

[15] Jia X., Xia Z., Gu B. (2013). Nonlinear viscoelastic multi-scale repetitive unit cell model of 3D woven composites with damage evolution. Int. J. Solids Struct..

[16] Tate J., Kelkar A., Beall G. (2006). Viscoelastic effects on fatigue behavior of braided composites. Asme Int. Mech. Eng. Congr. Expos..

[17] Zhai J., Kong X., Cheng S. (2020). A coupled multi-scale method for predicting the viscoelastic behavior of resin-based 3D braided composites. Mater. Des..

[18] Lin K., Hwang I. (1988). Thermo-viscoelastic response of graphite/epoxy composites. J. Eng. Mater. Technol..

[19] Muddasani M., Sawant S., Muliana A. (2010). Thermo-viscoelastic responses of multilayered polymer composites: Experimental and numerical studies. Compos. Struct..

[20] Li J., Yao X., Liu Y. (2010). Thermo-viscoelastic analysis of the integrated T-shaped composite structures. Compos. Sci. Technol..

[21] Seifert O., Schumacher S., Hansen A. (2003). Viscoelastic properties of a glass fabric composite at elevated temperatures: Experimental and numerical results. Compos. Part B-Eng..

[22] Hirsekorn M., Marcin L., Godon T. (2018). Multi-scale modeling of the viscoelastic behavior of 3D woven composites. Compos. Part A.

[23] Cai Y., Sun H. (2013). Thermo-viscoelastic analysis of three-dimensionally braided composites. Compos. Struct..

[24] Cai Y., Sun H. (2014). Dynamic response of thermo-viscoelasticity of three-dimensionally braided composites. J. Compos. Mater..

[25] Otero F., Oller S., Martínez X. (2015). Numerical homogenization for composite materials analysis. Comparison with other micro mechanical formulations. Compos. Struct..

[26] Karathanasopoulos N., Arampatzis G., Ganghoffer J. (2019). Unravelling the viscoelastic, buffer-like mechanical behavior of tendons: A numerical quantitative study at the fibril-fiber scale. J. Mech. Behav. Biomed..

[27] Karathanasopoulos N., Angelikopoulos P., Papadimitriou C. (2017). Bayesian identification of the tendon fascicle’s structural composition using finite element models for helical geometries. Comput. Methods Appl. Mech. Eng..

[28] Liu S., Ma N. (2005). Study on the thermal stress relaxation and constitutive equations of viscoelastic composite materials, part I: General theory. Acta Mater. Compos. Sin..

[29] Kalamkarov A. (1992). Composite and Reinforced Elements of Construction.

[30] Wei P., Zhang S., Wu Y. (1999). Correspondence principles and numerical methods of inverse integral transformation in viscoelastic mechanics. Adv. Mech..

[31] Zak A. (1968). Structural analysis of realistic solid propellant materials. J. Spacecr. Rockets.

[32] Press W., Flannery B., Teukolsky S., Vetterling W. (1992). Numerical Recipes in C: The Art of Scientific Computing.

[33] Zhai J., Cheng S., Zeng T., Wang Z., Fang D. (2017). Extended multiscale FE approach for steady-state heat conduction analysis of 3D braided composites. Compos. Sci. Technol..

[34] Zhai J., Zeng T., Xu G. (2017). A multi-scale finite element method for failure analysis of three-dimensional braided composite structures. Compos. Part B Eng..

[35] Ma N., Liu S. (2005). Study on the thermal stress relaxation and constitutive equations of viscoelastic composite materials, part II: Numerical simulation. Acta. Mater. Compos. Sin..

